# Conservative Ophthalmology

**Published:** 1911-09

**Authors:** F. Park Lewis

**Affiliations:** Buffalo, N. Y.


### BUFFALO MEDICAL JOURNAL

VOL. LXVII. SEPTEMBER, 1911. No. 2

### ORIGINAL COMMUNICATIONS

### Conservative Ophthalmology

By F. PARK LEWIS, M.D.
Buffalo, N. Y.

THE title of the paper which I have been asked to present, conservative ophthalmology, is apt to be a little misleading and to require an explanation. The thought which it suggests is that all ophthalmology should be conservative. While this is of course, true, there are certain conditions which exist or which may arise which demand prompt recognisation and immediate and proper attention or results follow which no subsequent treatment can remedy.

The importance of human vision is so great that the loss of even a portion of the sight which might have been saved, may so limit the usefulness of the person suffering such a loss as to change the whole course of his career. If for instance after an acute inflammatory condition of the eye, the patient emerges from the illness with but one half of his normal vision, or if after the healing of a corneal ulcer, absolutely normal vision for distance remains, but for the working range, by reason of the position of the corneal scars, is materially reduced, the general result would seem to be successful and the patient will have recovered but the limitation of vision will throughout his whole future life, prevent him from doing work which he might otherwise be qualified to do. It would limit his studies, if it occurred in childhood, it would prevent his adoption of any mechanical trade, requiring accurate vision, such as watch making, engraving or work of that kind. It would narrow his opportunities and circumscribe his possibilities in many ways.

Furthermore, injuries which are promptly and skilfully treated under certain conditions may leave almost absolutely normal sight, while delay or the application of improper methods may not only jeopardise the integrity of the vision of the affected eye, may not only result in blindness of that eye; but through sympathetic involvement of the fellow eye the result may be the absolute blindness of the patient. Happily modern methods have rendered such frightfully disastrous results far less common than they were a quarter of a century ago and still they occur often enough to make us realise that the employment of conservative measures such as are designed by promptitude and efficiency to save human sight are important enough to receive consideration.

I want, therefore, to emphasise some of those conditions in which the early recognisation is imperative if sight is to be saved. One of the commonest of these which we meet is ophthalmia neonatorum. Possibly so much has been said about this during the last few years that it will be necessary only to refer to it. There are some facts connected with it, however, which may bear repetition and concerning which I may say a few words. We all know how infection occurs in the earlier cases, but we forget sometimes that there is such a thing as a late infection. In other words, the mother having infectious discharges may at any time during the early life of the baby, may have the gonococci carried into the infants conjunctival sac. Even the general public is now becoming familiar with the nature of the infection, the methods of its prevention and the necessity of its early and prompt treatment. Still, when I say to you that in so progressive a center as that of Boston, of 116 cases, 114 occurred in the practice of physicians and only two under the care of midwives and of these 27 emerged with defective eyes and 9 were absolutely blind, we cannot say that the time has gone by when every one knows what to do for ophthalmia neonatorum.

If we are to control the disease of ophthalmia neonatorum, it must be by the concerted effort of the medical profession, the health authorities and the public. The following suggestions were made at the recent meeting of the American Medical Association to the department of hygiene and sanitation with a view of standardising methods of prevention which could be adopted in the several states. There seems to be little doubt that within the next few years, if activities continue, it will cease to be the serious menace that it has always been to the eyes of the new born.

"Blindness from birth infections has contributed one fourth to the population of all of the schools for the blind in the country. The existence of these infections is due to the failure on the part of the attendant, whether physician or midwife to use the proper preventive treatment for the child's eyes during the first few minutes after it is born.

Responsibility for this large amount of unnecessary blindness does not rest primarily upon the midwife and poor overworked doctor but it does rest upon society, which means every intelligent individual who knows what should be done and whose influence should be used to aid in the protection of these helpless babies.

The organised movement which originated in the American Medical Association, has now reached every state in the Union. Six states are gratuitously distributing the preventive remedy to every midwife and physician. The broader form of organisation is urged in which representatives from the medical profession, social service workers and others shall take upon themselves to see that right laws are enacted where they do not exist and that they shall be enforced where they have been enacted.

Mothers and prospective mothers are urged to join in the movement which may properly be carried on through women's clubs and other organisations of women.

Not only should the protective remedy be gratuitously distributed by the health department of the state and cities but it should be accompanied by pamphlets describing the nature of the birth infection, the manner in which it may be avoided and the necessity of immediate treatment when infection has occurred.

This should everywhere be made a reportable disease. Failure to report such cases should be followed by public prosecution of those who neglect to observe the laws. The publicity thus obtained is at once a warning and a deterrient.

It is perfectly evident that birth infections of the eyes can be stopped if organised and continuous efforts are made with this end in view. A concerted movement throughout the United States would be effective in saving the eyes of hundreds that would otherwise become blind."

The three essentials are a sanitary toilet, the use of a prophylactic and prompt and efficient treatment when the disease occurs. Under such conditions there practically need never be the loss of an eye from this disease.

The second condition which results with great frequency in lowered visual acuity is strabismus or squint. It formerly was not at all uncommon for physicians to advise the parents of a squinting child to wait until the boy was 16 or 17 years old before undertaking to have anything done for his relief. At six or seven years, I have often found it impossible to restore the inturned eye to full visual acuteness. Nature is exceedingly unwilling that there should be useless functions. Strabismus is almost invariably due to the fact that the eyes are organically malformed. It is the function of the two eyes to blend the images which they receive into a single picture like that obtained by the stereoscope. If the two sides of the human machine are mechanically different, it is impossible to force the two images to perfectly merge the one into the other. In the effort to accomplish this, the eye having the higher defect, or sometimes alternately, first one eye and then the other will be drawn in. When the two eyes are almost but not quite in line, double vision results. This is exceedingly uncomfortable. When the annoyance has persisted for a sufficient length of time, the eye which is turned in learns to suppress the image formed on that side. This is equivalent to saying that the visual center corresponding to the inturned eye has ceased to be active. When this dulling of a nerve center continues week after week for several years, the nerves in that portion of the brain cease to develop and we have in the corresponding eye, permanent amblopia. If on the other hand by correcting the focal difficulties so as to make the two eyes function normally, making the visual centers equally active on both sides, or if even we have an alternation of the squint so that one eye sees equally with the other, although its fellow may be turned in, we perpetuate the visual function and sight is preserved.

Whenever strasbismus is found, it should be placed under observation at the very earliest possible date. I have myself prescribed glasses for a squinting baby who is only five months old. In another instance, in that of an infant a year and thirteen days old, whose eyes converge badly, high hypermetropia was found in each eye, but differing in amount. The strong glasses which were necessary to make that baby see and to straighten its eyes and which were determined by means of the ophthalmoscope and the retinascope not only straightened the baby's eyes but gave the little sufferer such comfort, that two years later when the glasses had to be taken away in order to have the frames repaired, he begged to have them given back to him saying: "I want my eyes." That child will therefore, have normal vision in each eye, fitting him for any work that may be undertaken. Had the use of glasses been postponed until the eyes could not function normally the inevitable operation might have restored parallism, but it could not have restored vision and a correction so obtained would in all probability be followed by a divergence later in life.

Another condition which must have early recognisation which fortunately is of rare occurrence but when neglected produces frightful results, is glioma of the retina. This is supposed to have its origin at birth, but develops very slowly during the first two or three years of life, but at four or at latest five years of age, it can be seen by the naked eye. It is malignant growth arising from the retinal tissue. It slowly grows into the vitreous sometimes in whitish masses in the form of a feather duster, sometimes like flocculent masses, until by the time it has reached the posterior surface of the lens, it appears to the untrained eye like a cataract. When the light is thrown in by a mirror, milky masses may be seen in the deeper tissues of the eye. No pain exists with this until the eye ball becomes filled with the then rapidly developing substance. Then the pressure develops, tension increases and the child suffers periodically with attacks of pain. Later the pain becomes more intense and more frequent. Up to the present time, the growth is encapsulated into the eye and the removal of the eye which is the only known remedy will save the life of the child. But later, the growth breaks through the eye as it is sure to do and any operative measures are then likely to be too late. The orbital tissues have become involved and it rapidly passes to the brain. It grows like a mushroom from the orbit constituting the classical fungus hematodies. It penetrates the skull bursting its structure apart and destroys the life of the child with frightful suffering. Happily it occurs but once in ten thousand cases, but that one case may at any time come under the observation of any one of us and hard as it is to remove the eye from an apparently healthy child, it is the only thing to do and should be done at once.

Another phase of conservative ophthalmology is that which provides for the early recognisation and treatment of injuries of the eye and especially those in which foreign metallic bodies are carried into the eye ball. It is astonishing how excellent results may be obtained when immediate care is given. A little girl was brought to my office direct from school. She was sharpening a pencil with the blade of the knife toward the face. Another child coming from behind her pushed her head forward and the knife entered the eye. When she appeared in the office, the iris had prolapsed and was protruding through the wound. It was cut off with a clean incision, making a perfect iridectomy. The eye was treated antiseptically, bandaged and within a week, the wound was perfectly healed. Subsequent tests showed that her vision was absolutely normal. The blade fortunately had escaped the lens of the eye. Had she waited 24 hours, the probabilities are that the wound would have become infected and even if the eye had been saved, it would have been at the expense of an inflammatory action with plastic deposits which would have permanently ended its usefulness.

It goes almost without saying that a hurt directly involving the eye ball with a break which may even be superficial, in its structure, should be made as completely aseptic as possible at the earliest moment after the injury because it is the epithelium which is the barrier which germs find most difficult of entry; but it is not so commonly remembered that an early restoration of the tissues to as nearly a normal relation of their structure as possible within a few hours of the time at which the accident occurred, multiplies many times the chances of a successful issue. Twenty-four or thirty-six hours in which an open wound of the eye ball is left open to the entrance of septic germs may be quite enough to develop an infection which may result in loss of the eye.

### FOREIGN BODY IN THE VITREOUS.

A young man nineteen years old while driving a nail in a tank at his home in Titusville was suddenly conscious of a blow on his eye. A chip of metal had flown from the hammer or the nail striking the middle of the upper lid and puncturing the ball. The wound was evidently a large one as the eye was full of blood. The physician who was called applied cold compresses—but in 24 hours the upper lid was dark and edematous and the eye ball highly infected and exquisitely tender and painful. Twelve hours later the case coming under the care of another physician its gravity was immediately recognised and he was dispatched by the first train for special advice and treatment. He reached my office in the evening suffering acutely. As there was reason to believe that the foreign body was still in the eye the magnet was carefully employed. At the end of fifteen minutes a buzzing sound was heard, indicating that a metallic connection had been made and gradually and gently a piece of steel weighing 10 grains and of the shape of a long triangle enveloped in ropy pus was removed. There seemed little hope of saving the eye ball but the wound was irrigated with a two per cent. solution of protargol and the eye ball flooded with the same and the eye bandaged over an antiseptic pad. The wound healed rapidly and after two months treatment was wholly free from inflammation, only slightly smaller than its fellow. The extent of the inflammation and the duration of the treatment would both have been materially lessened if the sepsis could have been prevented.

### RUPTURE OF SCLERA WITH PROLAPSE OF IRIS RECOVERY WITH SUBSEQUENT DETACHMENT OF THE RETINA.

One of the most common causes of hurts of the eye ball is from a glancing blow on a nail which may either result in causing the nail itself to strike the eye or for a chip from the hammer or nail to do so. A business man undertook to do a little repairing at home. Unpractised in hammering, he struck the nail head a glancing blow with the result that the nail flew with great force striking the right eye causing great pain and immediately infusing it with blood. He was brought to my office within an hour when a rupture was found to exist at the corneal margin, the iris prolapsing. Without delay he was immediately placed in the office chair, the eye made as thoroughly aseptic as possible and the protracted iris excised. The clean wound healed with great rapidity and in a very few weeks normal vision was restored. After blows of this kind, however, the attachment of the margin of the retina to the sclera becomes insecure and three years later, while in California, he was suddenly taken with nose bleed which was protracted and profuse requiring radical measures for its relief. When it had been controlled, it was found that he had detachment of the retina at the point of the former wound with vision greatly reduced to merely a quantitated perception of light.

The condition is one which is more than likely to occur after a foreign body has been successfully removed from the vitreous even after several years have elapsed from the time of the original hurt and is a condition which must always be considered as a possibility.

But most important of any of the new kinds of work that have been done in regard to the saving of the eyes are those which enable us to use the giant magnet in retrieving bits of metal which have entered the deeper structures. The true nature of sympathetic ophthalmia is not yet understood, but we do know that foreign bodies left in the eye, not only jeopardise its own usefulness, but through sympathetic involvement, that of its fellow.

A foreign body within the eye, by means of special arrangement to be used with the *x*-ray can, in many instances be localised with almost absolute exactitude. The accompanying chart was made by Dr. L. Reu, whose skill in localising a foreign body by means of the *x*-ray has been tested many times.

In this particular instance, by a slight error in calculation, the foreign body was represented as being just outside of the eye ball in the vicinity of the ciliary region and above the horizontal meridian. It was known to be within the eye ball and was found at almost the exact point indicated.

Sometimes, however, by means of the giant magnet, the happiest results are obtained. An Italian workman came to me from Avon, some months ago. He had been engaged in cutting stone. There was every evidence that a foreign body had entered the eye. A small hole in the cornea, a puncture in the iris and a clot of blood in the vitreous which obscured a view of the eye ground were the three destructive points. He was taken to the

Charity Eye, Ear and Throat Hospital where we have a Haab magnet, operated by the electric light current and weighing about three hundred pounds. A bunch of keys thrown across the room, if they come within a foot of the point of the magnet, are immediately drawn to it. When this man was placed before the magnet, he immediately complained of a sense of pain, giving evidence that a metallic body was responding to the magnetic attraction. The manipulation was, therefore, very careful and gentle. At the end of ten minutes, a little bulging was seen at the edge of the iris. The magnet was thrown in such a way as to attract the body to the pupil. It could be seen approaching the edge and gradually slipping forward into the anterior chamber. A slight puncture was made in the edge of the cornea and a smaller magnetic point was produced and the tiniest piece of iron which had flown from the hammer was removed. It was certainly not more than one quarter of the size of the head of a pin and yet it was ample to have permanently destroyed the sight. This case was a most unusual one in the absolutely normal restoration of vision. Ramsey, the celebrated Scotch ophthalmologist in his classics on Injuries of the Eye, says that he has never seen a bit of metal recovered from the vitreous with perfect vision restored.

Another instance in which a bit of metal had remained in the eye for twenty years without exciting inflammatory reaction, about two months ago was found to be causing acute attacks of pain. These were controlled but occurred again for six weeks, at the end of which time the pain was constant. In this instance, the magnet gave no evidence whatever, of the existence of a foreign body although the *x*-ray picture located it with absolute precision. The eye was opened at the point in which the foreign body was shown to lie. The vitreous humor was found to have become fluid. The eye had of course been absolutely blind through all of these years. Still this strong magnetic attraction exercised no effect. The eye was removed and opened and the foreign body found so tightly impacted in a dense mass of cicatrical tissue that it still was not affected by this strong magnet and it was only when the foreign body had been exposed and placed almost at the tip of the magnet, that it was slowly drawn out of the tight grip in which these thickened structures held it.

Had the magnet been in use when the injury occurred, twenty years ago, there is no reason to doubt that the eye ball, at least, could have been saved, even if the vision had been lost.

454 Franklin Street.

